# Supplementary material for: Highlighting In Vitro the Role of Brain-like Endothelial Cells on the Maturation and Metabolism of Brain Pericytes by SWATH Proteomics
Source: Cells. 2023 Mar 25;12(7):1010. doi: 10.3390/cells12071010 (PMC10093307; doi:10.3390/cells12071010)
Supplement: Supplementary file 1 [file cells-12-01010-s001.zip › cells-2279944 - Supplementary Materials fro Proof .pdf]

cells

MDPI

Supplementary Material

Highlighting In Vitro the Role of Brain-Like Endothelial Cells on the Maturation and Metabolism of Brain Pericytes by SWATH Proteomics

Camille Menaceur [1], Johan Hachani [1], Shiraz Dib [1], Sophie Duban-Deweer [1], Yannis Karamanos [1], Fumitaka Shimizu [2], Takashi Kanda [2], Fabien Gosselet [1], Laurence Fenart [1] and Julien Saint-Pol [1,*]

[1] Univ. Artois, UR 2465, Blood-Brain Barrier Laboratory (LBHE), F-62300 Lens, France
[2] Department of Neurology and Clinical Neuroscience, Graduate School of Medicine, Yamaguchi University, Ube, 755-8505 Japan
* Correspondence: julien.saintpol@univ-artois.fr

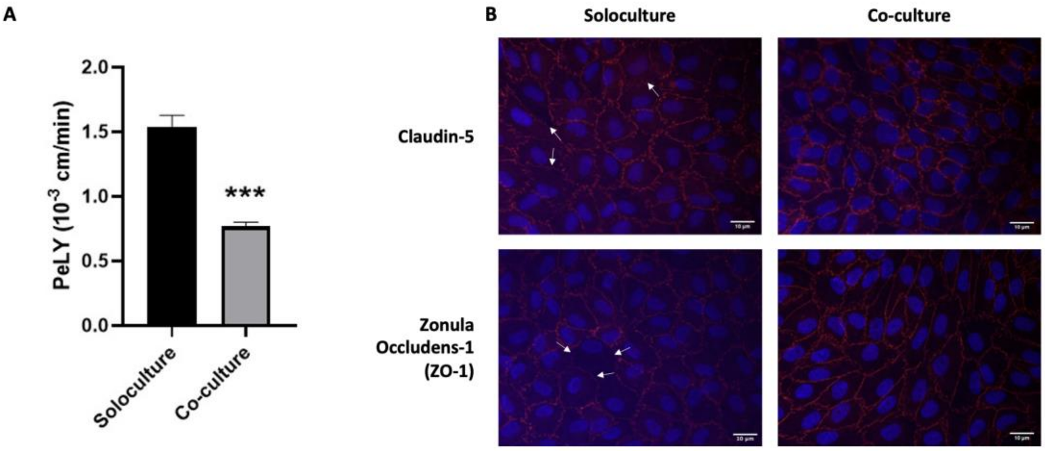

Figure S1. Modeling of the human BBB in vitro [29,30]. (**A**). Permeability assay to Lucifer yellow (LY) in ECs solocultured (Soloculture) or cocultured with hBPs for 6 days (Coculture). ***: $p < 0.001$ for t-test. (**B**) Immunostaining of two main proteins involved in tight junctions, Claudin-5 and ZO-1. White arrows show leakages or thin labeling in Soloculture compared with Coculture.

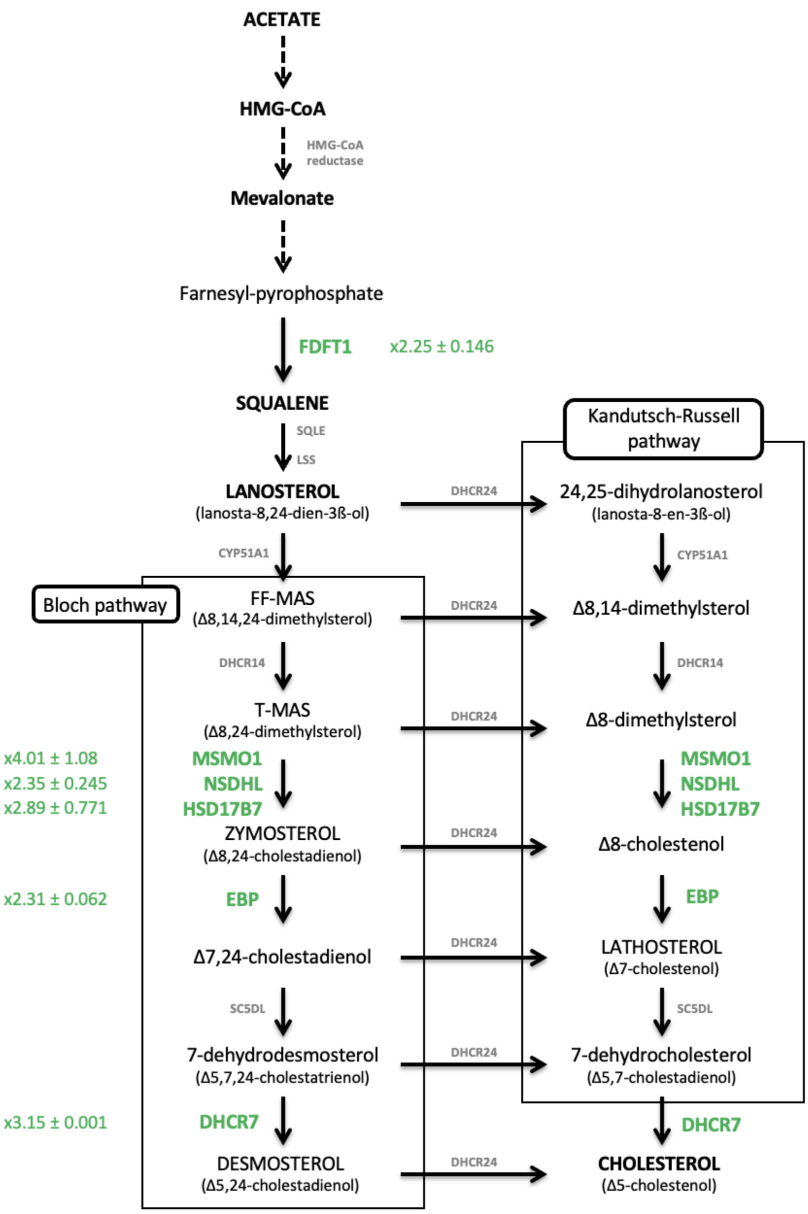

Figure S2. Enzymes of Bloch and Kandutsch-Russell cholesterol synthesis pathways are upregulated in hBP-coc. Each enzyme enriched in hBP-coc is notated in green with the corresponding SWATH quantification. Acronyms: CYP51A1: Lanosterol 14-alpha demethylase; DHCRs: desoxycholesterol reductases; EBP: 3-beta-hydroxysteroid-Delta(8),Delta(7)-isomerase; FDFT1: Squalene synthase; HMG-CoA: 3-hydroxy-3-methylglutaryl-coenzyme A; HSD17B7: 3-keto-steroid reductase/17-beta-hydroxysteroid dehydrogenase 7; LSS: lanosterol synthase; MSMO1: Methylsterol monooxygenase 1; NSDHL: Sterol-4-alpha-carboxylate 3-dehydrogenase, decarboxylating; SCSDL: Lathosterol oxygenase; SQLE: Squalene monooxygenase.

Table S3. List of the 51 proteins enriched in solocultured hBP (hBP-solo). Only quantified proteins with *p*-value < 0.01 and mean of FC >2 have been selected as significant candidates.

Accession Number	Gene Name	Detailed Name	Mean [a]	S.D.
P02452	COL1A1	Collagen alpha-1(I) chain	13.8	1.62
P08123	COL1A2	Collagen alpha-2(I) chain	9.92	0.910
P02461	COL3A1	Collagen alpha-1(III) chain	9.89	0.257
Q01995	TAGLN	Transgelin	7.03	0.979
P24844	MYL9	Myosin regulatory light polypeptide 9	4.67	0.737
Q9Y617	PSAT1	Phosphoserine aminotransferase	4.19	0.098
P15924	DSP	Desmoplakin	3.62	0.07
P09493	TPM1	Tropomyosin alpha-1 chain	3.57	1.12
P42566	EPS15	Epidermal growth factor receptor substrate 15	3.51	0.814
Q05682	CALD1	Caldesmon	3.43	0.973
P24821	TNC	Tenascin	3.42	1.09
Q5EB52	MEST	Mesoderm-specific transcript homolog protein	3.29	0.613
P20908	COL5A1	Collagen alpha-1(V) chain	3.27	0.398
Q8WX93	PALLD	Palladin	3.22	0.481
P78330	PSPH	Phosphoserine phosphatase	3.21	0.518
Q71U36	TUBA1A	Tubulin alpha-1A chain	3.18	0.393
Q96IZ0	PAWR	PRKC apoptosis WT1 regulator protein	3.17	0.641
O15460	P4HA2	Prolyl 4-hydroxylase subunit alpha-2	3.16	0.345
P34741	SDC2	Syndecan-2	3.13	1.343
P08243	ASNS	Asparagine synthetase [glutamine-hydrolyzing]	2.99	0.321
Q14195	DPYSL3	Dihydropyrimidinase-related protein 3	2.78	0.157
P00533	EGFR	Epidermal growth factor receptor	2.78	0.550
P14174	MIF	Macrophage migration inhibitory factor	2.76	0.428
Q9UGI8	TES	Testin	2.74	0.33
P05997	COL5A2	Collagen alpha-2(V) chain	2.70	0.007
P13611	VCAN	Versican core protein	2.70	0.381
P35555	FBN1	Fibrillin-1	2.67	0.289
Q15147	PLCB4	1-phosphatidylinositol 4,5-bisphosphate phosphodiesterase beta-4	2.67	0.138
P02751	FN1	Fibronectin	2.65	0.307
Q99584	S100A13	Protein S100-A13	2.60	0.245
O60701	UGDH	UDP-glucose 6-dehydrogenase	2.58	0.15
P31949	S100A11	Protein S100-A11	2.54	0.064
Q96JY6	PDLIM2	PDZ and LIM domain protein 2	2.47	0.077
Q16555	DPYSL2	Dihydropyrimidinase-related protein 2	2.46	0.154
O43175	PHGDH	D-3-phosphoglycerate dehydrogenase	2.45	0.065
Q9H910	HN1L	Jupiter microtubule associated homolog 2	2.42	0.336
P18206	VCL	Vinculin	2.37	0.139
Q59GN2	RPL39P5	Putative 60S ribosomal protein L39-like 5	2.34	0.414
P00338	LDHA	L-lactate dehydrogenase A chain	2.31	0.135
Q9H1E3	NUCKS1	Nuclear ubiquitous casein and cyclin-dependent kinase substrate 1	2.31	0.714
P37802	TAGLN2	Transgelin-2	2.30	0.177
P60660	MYL6	Myosin light polypeptide 6	2.28	0.397
Q07954	LRP1	Prolow-density lipoprotein receptor-related protein 1	2.25	0.076
Q14192	FHL2	Four and a half LIM domains protein 2	2.24	0.336
P40123	CAP2	Adenylyl cyclase-associated protein 2	2.21	0.124
P21333	FLNA	Filamin-A	2.19	0.057
O00193	SMAP	Small acidic protein	2.18	0.431
Q15582	TGFBI	Transforming growth factor-beta-induced protein ig-h3	2.13	0.144
Q13642	FHL1	Four and a half LIM domains protein 1	2.12	0.356
Q9NR12	PDLIM7	PDZ and LIM domain protein 7	2.08	0.000
Q14847	LASP1	LIM and SH3 domain protein 1	2.06	0.189

[a] Mean from the two independent SWATH analysis.

Table S4. List of the 90 proteins enriched in co-cultured hBP (hBP-coc). Only quantified proteins with p-value < 0.01 and mean of FC >2 have been selected as significant candidates.

Accession Number	Gene Name	Detailed Protein Name	Mean [a]	S.D.
P21980	TGM2	Protein-glutamine gamma-glutamyltransferase 2	7.57	0.21
Q13201	MMRN1	Multimerin-1	7.35	1.30
P05362	ICAM1	Intercellular adhesion molecule 1	6.97	0.84
P08572	COL4A2	Collagen alpha-2(IV) chain	4.34	1.00
P35625	TIMP3	Metalloproteinase inhibitor 3	4.07	0.29
P00750	PLAT	Tissue-type plasminogen activator	4.03	0.64
P52566	ARHGDIB	Rho GDP-dissociation inhibitor 2	4.03	0.77
Q15800	MSMO1	Methylsterol monooxygenase 1	4.01	1.08
O00622	CYR61	CCN family member 1	3.92	0.11
O94919	ENDOD1	Endonuclease domain-containing 1 protein	3.92	0.94
O00767	SCD	Stearoyl-CoA desaturase	3.91	0.57
P01130	LDLR	Low-density lipoprotein receptor	3.47	0.40
Q13451	FKBP5	Peptidyl-prolyl cis-trans isomerase FKBP5	3.44	0.22
Q8WTV0	SCARB1	Scavenger receptor class B member 1	3.37	1.632
P36269	GGT5	Glutathione hydrolase 5 proenzyme	3.28	0.739
Q9UBM7	DHCR7	7-dehydrocholesterol reductase	3.15	0.001
O95864	FADS2	Acyl-CoA 6-desaturase	3.13	0.514
O75600	GCAT	2-amino-3-ketobutyrate coenzyme A ligase, mitochondrial	3.05	0.656
Q01628	IFITM3	Interferon-induced transmembrane protein 3	3.01	0.099
Q709F0	ACAD11	Acyl-CoA dehydrogenase family member 11	2.92	0.093
P30519	HMOX2	Heme oxygenase 2	2.89	0.387
P56937	HSD17B7	3-keto-steroid reductase/17-beta-hydroxysteroid dehydrogenase 7	2.89	0.771
Q9Y276	BCS1L	Mitochondrial chaperone BCS1	2.85	0.287
Q7Z7K6	CENPV	Centromere protein V	2.76	0.177
O94875	SORBS2	Sorbin and SH3 domain-containing protein 2	2.75	0.064
P11169	SLC2A3	Solute carrier family 2, facilitated glucose transporter member 3	2.75	0.061
O14746	TERT	Telomerase reverse transcriptase	2.74	0.369
Q9UDW1	UQCR10	Cytochrome b-c1 complex subunit 9	2.73	0.759
P49327	FASN	Fatty acid synthase	2.66	0.024
Q8N4Q1	CHCHD4	Mitochondrial intermembrane space import and assembly protein 40	2.63	0.050
P10301	RRAS	Ras-related protein R-Ras	2.56	0.272
P05121	SERPINE1	Plasminogen activator inhibitor 1	2.55	0.121
P02792	FTL	Ferritin light chain	2.53	0.296
Q92947	GCDH	Glutaryl-CoA dehydrogenase, mitochondrial	2.53	0.328
O75521	ECI2	Enoyl-CoA delta isomerase 2	2.51	0.018
P21589	NT5E	5'-nucleotidase	2.50	0.050
Q9Y619	SLC25A15	Mitochondrial ornithine transporter 1	2.47	0.071
Q6PI48	DARS2	Aspartate--tRNA ligase, mitochondrial	2.47	0.345
Q12931	TRAP1	Heat shock protein 75 kDa, mitochondrial	2.47	0.141
P11387	TOP1	DNA topoisomerase 1	2.43	0.000
P14543	NID1	Nidogen-1	2.43	0.140
Q9NZ45	CISD1	CDGSH iron-sulfur domain-containing protein 1	2.41	0.049
Q9NX58	LYAR	Cell growth-regulating nucleolar protein	2.40	0.104
P05067	APP	Amyloid-beta precursor protein	2.36	0.739
Q9Y3A6	TMED5	Transmembrane emp24 domain-containing protein 5	2.36	0.143
Q15738	NSDHL	Sterol-4-alpha-carboxylate 3-dehydrogenase, decarboxylating	2.35	0.245
P08962	CD63	CD63 antigen	2.33	0.124
Q8TED0	UTP15	U3 small nucleolar RNA-associated protein 15 homolog	2.32	0.500
Q15125	EBP	3-beta-hydroxysteroid-Delta(8),Delta(7)-isomerase	2.31	0.062
Q9H936	SLC25A22	Mitochondrial glutamate carrier 1	2.31	0.567
Q9UJZ1	STOML2	Stomatin-like protein 2, mitochondrial	2.29	0.304
Q02218	OGDH	2-oxoglutarate dehydrogenase, mitochondrial	2.28	0.191

P42785	PRCP	Lysosomal Pro-X carboxypeptidase	2.27	0.029
Q9Y697	NFS1	Cysteine desulfurase, mitochondrial	2.26	0.042
P13612	ITGA4	Integrin alpha-4	2.26	0.379
P60602	ROMO1	Reactive oxygen species modulator 1	2.26	0.043
Q9Y3E5	PTRH2	Peptidyl-tRNA hydrolase 2, mitochondrial	2.26	0.301
P37268	FDFT1	Squalene synthase	2.25	0.146
P15151	PVR	Poliovirus receptor	2.25	0.121
Q9UNX4	WDR3	WD repeat-containing protein 3	2.24	0.122
Q9BV79	MECR	Enoyl-[acyl-carrier-protein] reductase, mitochondrial	2.22	0.231
O43819	SCO2	Protein SCO2 homolog, mitochondrial	2.21	0.353
P61604	HSPE1	10 kDa heat shock protein, mitochondrial	2.20	0.055
Q07021	C1QBP	Complement component 1 Q subcomponent-binding protein, mitochondrial	2.20	0.010
P30405	PPIF	Peptidyl-prolyl cis-trans isomerase F, mitochondrial	2.19	0.436
P00367	GLUD1	Glutamate dehydrogenase 1, mitochondrial	2.16	0.036
O75616	ERAL1	GTPase Era, mitochondrial	2.15	0.182
O00560	SDCBP	Syntenin-1	2.14	0.221
P13473	LAMP2	Lysosome-associated membrane glycoprotein 2	2.14	0.103
Q03405	PLAUR	Urokinase plasminogen activator surface receptor	2.14	0.374
Q9Y3A4	RRP7A	Ribosomal RNA-processing protein 7 homolog A	2.11	0.011
P02794	FTH1	Ferritin heavy chain	2.11	0.058
P29279	CTGF	CCN family member 2	2.11	0.042
Q14684	RRP1B	Ribosomal RNA processing protein 1 homolog B	2.10	0.195
Q9BQ39	DDX50	ATP-dependent RNA helicase DDX50	2.09	0.084
Q9BUB7	TMEM70	Transmembrane protein 70, mitochondrial	2.08	0.269
Q9NYP7	ELOVL5	Elongation of very long chain fatty acids protein 5	2.08	0.066
P08574	CYC1	Cytochrome c1, heme protein, mitochondrial	2.07	0.037
P10515	DLAT	Dihydrolipoyllysine-residue acetyltransferase component of pyruvate dehydrogenase complex, mitochondrial	2.06	0.091
P17096	HMGA1	High mobility group protein HMG-I/HMG-Y	2.06	0.057
Q9H2U2	PPA2	Inorganic pyrophosphatase 2, mitochondrial	2.06	0.101
Q13740	ALCAM	CD166 antigen	2.06	0.107
Q16762	TST	Thiosulfate sulfurtransferase	2.05	0.170
Q12929	EPS8	Epidermal growth factor receptor kinase substrate 8	2.03	0.081
Q6ZRP7	QSOX2	Sulfhydryl oxidase 2	2.03	0.094
Q8IVS2	MCAT	Malonyl-CoA-acyl carrier protein transacylase, mitochondrial	2.03	0.124
Q9Y221	NIP7	60S ribosome subunit biogenesis protein NIP7 homolog	2.02	0.231
O95573	ACSL3	Long-chain-fatty-acid--CoA ligase 3	2.02	0.049
Q13595	TRA2A	Transformer-2 protein homolog alpha	2.00	0.028
P10809	HSPD1	60 kDa heat shock protein, mitochondrial	2.00	0.007

[a] Mean from the two independent SWATH analysis.

Supplementary Methods

S1- Evaluation of endothelial cells (ECs) permeability

Filters containing monolayers of non-differentiated (Soloculture) or differentiated ECs (Coculture) were transferred to wells of 12-well plates without cells and containing 1.5 mL of Ringer-HEPES solution (150 mM NaCl, 5.2 mM KCl, 2.2 mM $CaCl_2$, 0.2 mM $MgCl_2$-$6H_2O$, 6 mM $NaHCO_3$, 5 mM HEPES, 2.8 mM glucose; pH: 7.4) at 37 °C. In the filter, the culture medium was replaced with 0.5 mL RH containing 50 µM Lucifer yellow (LY) for 60 min. Lucifer yellow is then quantified using the Synergy H1 hybrid reader fluorometer (BioTech) in each aliquot of the lower, upper, and initial solution compartments. The principle of clearance is used as X/C where X is the amount of Y detected in the lower compartment and C is the concentration in the upper compartment. The value of the clearance is plotted against time and the slope of the line obtained gives the value of PSt (filter permeability + Matrigel™ + ECs X filter surface) and PSf (filter permeability + Matrigel™ X filter surface) in order to calculate PSe (endothelial permeability x filter surface) according to the formula: $\frac{1}{PSe} = \frac{1}{PSt} - \frac{1}{PSf}$. The endothelial permeability coefficient (Pe) is determined by dividing PSe by the filter surface (S=1.12 cm^2).

S2- Immunostaining for tight junctions (-associated) proteins

Solocultured or cocultured ECs on filters are fixed with 4% paraforamdehyde (Thermoscientific, J61984) for 10 minutes and rinsed three times with PBS-CMF. The filters are then cut and permeabilized in Triton X-100 0,1% and rinsed several times with PBS-CMF. The non-specific sites werw saturated with Sea Blocking Buffer (SBB, ThermoScientific 37527) for 30 min. Cells were then incubated for 1 h at room temperature with primary antibodies against Claudin-5 (Rabbit polyclonal, 1/100, Invitrogen 34600) or ZO-1 (Rabbit polyclonal, 1/200, Invitrogen 617300 diluted in PBS-CMF supplemented with 2% Goat Serum Donnor Herd (GS, Sigma, G6767). Three rinses were performed in PBS-CMF-GS 2% before incubating the cells with the fluorochrome-coupled secondary antibody (dilution in PBS-GS 2%) for 1 h at room temperature. Filters were rinsed three times with PBS-CMF and mounted on slides in Prolong® solution, a fluorescence stabilizing agent (Invitrogen, P36962). Slides were then stored at 4 °C. Labeled cells were observed and pictured under a Leica fluorescence microscope (DMi8, Leica Microsystems) associated with Leica LASX software (Leica Microsystems).
